# Acute Awake Fiberoptic Intubation in the ICU in a Patient with Limited Mouth Opening and Hypoxemic Acute Respiratory Failure

**DOI:** 10.1155/2019/6421910

**Published:** 2019-10-23

**Authors:** Kjartan E. Hannig, Rasmus W. Hauritz, Christian Jessen, Anders M. Grejs

**Affiliations:** ^1^Department of Anesthesiology, Kolding Hospital, 6000 Kolding, Denmark; ^2^Department of Anesthesiology, Horsens Hospital, 8700 Horsens, Denmark; ^3^Department of Intensive Care Medicine, Aarhus University Hospital, 8200 Aarhus N, Denmark

## Abstract

The incidence and survival of patients with head-and-neck cancer have been on the increase for decades. Following surgery or radiation therapy, complications such as difficult airways may evolve. These difficult airways may be unique and not manageable with conventional intubation methods as well as video laryngoscopes. Acute awake fiberoptic intubation may be a feasible option also for urgent emergency airway management of known difficult airways. The “cannot intubate–cannot oxygenate” (CI–CO) situation has to be avoided at all costs, since emergency cricothyrotomy has a fail ratio of more than 50% when performed by an anesthesiologist.

## 1. Introduction

In Denmark, the incidence of head-and-neck cancer (HNC) has more than doubled since 1980 and the relative 5-year survival has risen from 49% to 72% [1]. Previous HNC surgery or radiation therapy is a strong predictor of a future difficult airway [2]. Of all patients undergoing an emergency cricothyrotomy due to a “cannot intubate–cannot oxygenate” (CI–CO) scenario, 75% went through HNC surgery at some point prior to the event [2]. Hence, it is likely that the proportion of patients with potentially difficult airways will increase in hospitals without ear-nose-throat (ENT) expertise, with acute symptoms requiring immediate treatment. Our hypothesis is that all hospitals managing patients in acute respiratory failure should not only have backup plans for *unexpected* difficult airways but also for the *expected *ones [3]. This optimally includes skills in awake fiberoptic intubation (FOI), which preserves spontaneous breathing and thus can prevent the risk of critical desaturation or a CI–CO situation [4].

## 2. Case Presentation

Written informed patient consent for publication was obtained. Approval from The Central Denmark Region Committee on Health Research Ethics was unwarranted.

A 71-year-old man (height 166 cm, weight 60 kg) with previous HNC, presented in the emergency room due to fever and increasing dyspnea during the last 4 days.

**Figure 1:**
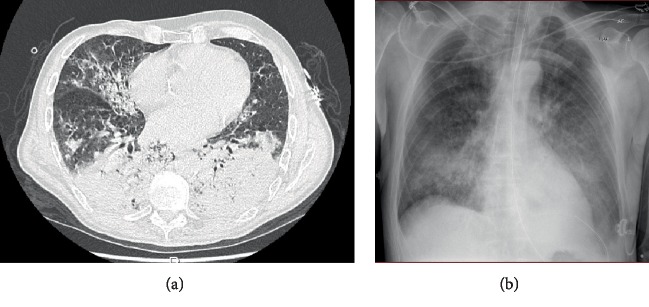
Computed tomography of lungs at admission (a) and chest X-ray immediately after intubation (b) showing massive bilateral pneumonia.

On arrival, the patient was fully alert and oriented. Respiratory rate was 40 breaths/min, peripheral saturation was 80% with 10 L/min oxygen delivered on a Hudson mask, blood pressure was 91/64 mmHg, heart rate was 110 beats/min, and temperature was 38.5°C. The Hudson mask was replaced with a reservoir mask with an oxygen flow of 15 L/min. A computed tomography scan including intravenous contrast was performed, which showed bilateral pneumonia and excluded pulmonary embolism (Figure 1). The patient was transferred to the intensive care unit (ICU), where high-flow nasal oxygen (HFNO) was administered with a flow of 60 L/min and a fraction of inspired oxygen (FiO_2_) of 100%. However, this was quickly amended to noninvasive ventilation (NIV) with a positive end expiratory pressure of 12 cm H_2_O and a FiO_2_ of 100%. Oxygenation remained unacceptably low (Table 1). Accordingly, the decision to intubate was made.

In 1999, the patient's tonsil cancer had been treated with radiation therapy. Unfortunately, this caused mandibular osteoradionecrosis, which had necessitated hemi-mandibulectomy and comprehensive reconstruction surgery (free fibula flap combined with a pedicled pectoralis major myocutaneous flap) on the right side in 2003 and on the left side in 2015. The surgeries were performed following awake FOI and surgical tracheostomy. In 2014, at another hospital, a laparoscopic cholecystectomy necessitated intubation, which was attempted after the induction of anesthesia. A nasal approach utilizing a video laryngoscope (VL) with a hyper angulated blade (McGrath™ MAC X-Blade™, Medtronic, MN) failed, and the esophagus was intubated twice. This was attributed to the extreme limited mouth opening, which made visualization of the relevant structures impossible. Further nasal attempts were stopped due to bleeding, and intubation finally succeeded orally with a fiberscope. Since this episode, only awake FOI attempts had been performed.

Other comorbidities included arterial hypertension (treated with an Angiotensin-II-receptor-blocker), chronic jaw pain (treated with oral morphine 40 mg/day) and a history of smoking for 28 years (½ pack of pipe tobacco/week). Despite his comorbidities, he was physically active and played golf on a weekly basis.

**Figure 2:**
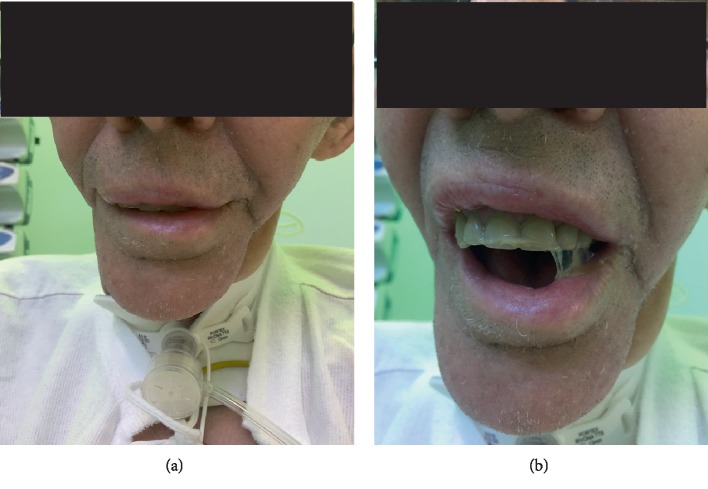
The patient 3 weeks after admission—after surgical tracheostomy (a and b). Maximal mouth opening of 10 mm is presented (b).

His airway examination revealed a mouth opening of 10 mm and neck movement <80° (due to the bilateral fibrous myocutaneous flaps), among other factors (Figure 2 and Table 2). Most important, however, was the history of previous neck radiation and difficult intubation.

In the ICU, the patient was placed in the upright sitting position to provide airway patency and to ease his respiratory efforts. Glycopyrrolate 0.2 mg IV (Meda, Solna, Sweden) was administered once. Sedation was titrated slowly with midazolam 0.5 mg IV (Hameln, Hameln, Germany) and *S*-ketamine 2.5 mg IV (Pfizer, Ixelles, Belgium), ensuring slight sedation but cooperation and responsiveness corresponding to a level 2 on the Ramsey Sedation Scale. Airway topicalization was initiated with lidocaine 2% (Mylan, Canonsburg, PA) 4 mL nebulized using the NIV machine. This was done twice at 5-minute intervals (total of 160 mg). Lidocaine 10% (AstraZeneca, Södetälje, Sweden) was sprayed onto the base of the tongue and posterior pharynx, subsequently deeper on each of 3 times at 3-4-minute intervals (total of 90 mg). Transtracheal injection of lidocaine 4% 1 mL was also administered (total of 40 mg). As potential backup plans, the right nasal cavity was prepared with lidocaine 2% with epinephrine 5 *µ*g/mL, using both a mucosal atomizing device (MAD Nasal™, Teleflex, Wayne, PA) and lidocaine soaked ribbon gauze (total of 20 + 60 mg), and infiltration in front of the cricothyroid membrane with 3 mL (total of 60 mg) was performed. After 12 minutes of preparation, the patient was changed back from NIV to HFNO and an uneventful oral FOI was performed with a reinforced LMA Fastrach™ (Teleflex, Waine, PA) endotracheal tube with an internal diameter of 7.5 mm using the aScope™ Regular (Ambu, Ballerup, Denmark). The FOI in itself took less than 2 min. Correct tube placement was confirmed visually and with capnography, Propofol-infusion was initiated (70 *µ*g/kg/min) and the patient was connected to the ventilator. Five weeks of ventilator therapy followed. Eight days after admission, a surgical tracheostomy was performed at the ENT facility of another hospital. The patient was discharged to his home 8 weeks after admission.

## 3. Discussion

National guidelines on the management of difficult airways have been published in numerous countries including the USA, the United Kingdom and Canada [3]. The ultimate goal of all airway management is to avoid a CI–CO scenario, since emergency cricothyrotomy performed by anesthesiologists fails in more than 50% of these situations [2, 5].

For the *unexpected difficult airway* focus should be on prediction and planning, as to decrease the incidence of its occurrence in the anesthetized and paralyzed patient [3]. Although several bedside tests can be used to anticipate the likelihood of a difficult airway, unfortunately no clinical finding can reliably exclude a difficult airway [6, 7]. If difficulties arise after the induction of anesthesia, rescue plans and algorithms should focus on oxygenation and not just center around intubation [3]. Emphasis should be on both technical and non-technical skills [3].

For the *expected difficult airway* an awake airway securing technique may be the safest option, since it facilitates airway patency, oxygenation and protection against aspiration [3, 4]. Awake intubation is considered a safe option when difficulties with intubation are anticipated and backup plans for maintaining oxygenation are expected to be difficult (facemask ventilation, supraglottic device (SGD) placement and/or emergency cricothyrotomy) [3, 4]. The presence of obstructing airway pathology, risk of rapid desaturation (e.g. respiratory failure) and increased risk of aspiration all favor an awake approach [3, 8].

The first awake FOI was described by Murphy in 1967 [9], and since the 1970's awake FOI has been the “gold standard” for managing anticipated difficult airways [3, 4, 10]. In recent years, awake VL intubation has been described with similar success rates as awake FOI [10]. In cases of uniquely altered anatomy (e.g., limited mouth opening, HNC in the oral cavity or upper airway and previous radiation), insertion of the VL blade with free sight may, however, be impossible [11]. In our case, use of a VL had previously been impossible due to the extremely limited mouth opening. Usually, the following minimal mouth opening must be present for reliable airway maneuvers: Macintosh intubation > 25 mm, SGD usage > 25 mm and VL intubation > 20 mm [8, 12, 13]. Nevertheless, successful use of these devices in patients with less mouth opening has been reported [14].

Experts disagree as to whether awake FOI skills should be mastered by every anesthesiologist or only restricted to practitioners who deal with these difficult airways on a daily basis [15]. A survey among US anesthesiologists in 2003 found that only 59% reported to have skills in FOI [16]. Opportunities to acquire FOI skills in the operating room have decreased since the introduction of the VL in clinical practice in 2002. An acceptable level of technical skills may be acquired after performing 10 FOI's in general anesthesia and 15–20 on awake patients [8, 17], but true expertise probably requires more training. This necessitates heavy weighting of trainees performing supervised FOI's, optimally as part of a routine institutional practice [4]. New airway simulators consisting of components including replica video bronchoscopes, and desktop sensors can create virtual patients with difficult airways. This possibly may accelerate obtaining FOI skills, even though transferability of these skills to performance in real life patients with difficult airways has not yet been shown.

In a recent review on elective awake FOI protocols in the operating room, no specific strategy regarding premedication, local anesthesia, or sedation could be declared superior to the others [5]. In the critically ill patient, with minimal physiological reserves, airway obstruction (due to over-sedation) and respiratory or circulatory collapse would be dangerous. Sedation should thus be kept to a minimum (or no sedation at all) and nonopioid medication including ketamine may be the safest choice. In the end, familiarity of the anesthesiologist performing the FOI with their drug of choice is probably the most important point [5].

The disadvantage of FOI is that blood and secretions in the airway or at the tip of the fiberscope may obscure the vision. A thin suctioning catheter can be placed nasally in the pharynx for continuous suctioning and cautious low-flow oxygen through the working channel of the fiberscope can be administered to continuously flush the tip. Both of these techniques were prepared for, but not needed since visibility was obtained immediately. Some argue that FOI is extremely time consuming. However, with the right preparations undertaken simultaneously, this may not be true [8, 11].

Other theoretical options would have been classical blind techniques used for decades, such as blind nasal [18], retrograde [19] or light wand intubation [20]. Nevertheless, these are increasingly becoming a lost art and even if local expertise exists, success rates are not necessarily acceptable [21]. It is possible that an oral retromolar approach with a rigid intubating stylet (e.g., Bonfils™ [22]) or a nasal approach with the newer rigid video-intubating stylet (Trachway™ [23]) may have worked, but equipment and expertise are not available in all departments. Placing the patient on extracorporeal membrane oxygenation (ECMO) before performing a surgical tracheostomy has been reported in extreme circumstances [24]. However, besides potential risk of major hemorrhaging, highly specialized equipment and expertise in thoracic and ENT surgery are required.

The airway plan was an awake oral FOI. It was preferable to avoid the nasal route, since it was probably necessary to decrease the tube size and since nasal bleeding would have been disastrous. Accordingly, an awake nasal FOI was the second choice. As backup plan in hospitals *with* ENT surgical expertise an awake surgical tracheostomy would have been a viable option or could have represented the primary choice instead of the awake FOI. In hospitals *without* ENT surgical expertise (as in this case), an awake percutaneous dilatational tracheostomy (if experienced intensivists are present) or an awake cricothyrotomy could have been the potential backup plan, keeping in mind that these procedures may be technically very challenging, when performed in local anesthesia in the critically ill patient on spontaneous breathing. The last option in either case would have been an emergency cricothyrotomy performed under adequate anesthesia and full relaxation.

Once the transcricothyroidal local anesthetic was given, using an adequately sized plastic cannula instead of the small-bore needle and leaving it on site as a backup for cricothyrotomy would have been a possibility, providing access for temporary emergency jet oxygenation or for placement of a guidewire in the trachea in connection with Seldinger based cricothyrotomy sets.

## 4. Conclusion

This case illustrates that patients with difficult airways may present at hospitals without ENT expertise, requiring urgent intubation, which is not manageable with conventional methods as well as VL. Optimal skills for performing an awake FOI should be present around the clock. As backup, an awake tracheal access can be considered: awake surgical tracheostomy (if ENT surgical expertise is present), awake percutaneous dilatational tracheostomy (if specially experienced intensivists are present) or awake cricothyrotomy (if neither ENT surgeons or intensivists are present). These methods may be the only options to avoid emergency cricothyrotomy, which has an unacceptable high failure rate when performed by an anesthesiologist in a CI–CO scenario.

## Figures and Tables

**Table 1:** Arterial blood gas values at different times before intubation.

	Reservoir-mask (ER)	HFNO (ICU)	NIV (ICU)
pH (7.35-7.45)	7.29	7.33	7.34
Base excess (−3–3 mmol/L)	−10.0	−8.1	−8.3
pO_2_ (11.1–14.4 kPa)	8.6	5.8	7.8
Saturation (%)	88	74	88
pCO_2_ (4.7–6.4 kPa)	4.3	4.3	4.0
Lactate (0.5–1.6 mmol/L)	5.7	6.1	5.9

Abbreviations: ER, emergency room; HFNO, high-flow nasal oxygen; ICU, intensive care unit; NIV, noninvasive ventilation.

**Table 2:** Some data on predicted difficulties with intubation and backup plans for oxygenation. Highlighted (with a “+”), which of these features our patient presented [3, 6, 7].

Predictors of difficult intubation		Predictors of difficult backup plans for oxygenation	
*Predictors of difficult direct laryngoscopy*		*Predictors of difficult facemask ventilation*	

History of difficult intubation	+	History of neck radiation	+
Limited mouth opening (interincisor gap)	+	History of snoring or obstructive sleep apnea	+
Modified Mallampati Score 3 + 4	+	Obesity	
Limited cervical spine mobility	+	Older age	+
Upper lip bite test	+	Male sex	+
Limited mandibular protrusion	+	Full beard	
Retrognathia	+	Lack of teeth	+
Short thyromental distance	+	Modified Mallampati Score 3 + 4	+
		Limited mandibular protrusion	+
		Short thyromental distance	+

*Predictors of difficult video laryngoscopy*		*Predictors of difficult supraglottic device use*	

Neck pathology (e.g., scars, neck radiation, mass, thick neck)	+	Glottic, supraglottic or subglottic pathology (e.g., neck radiation)	+
Cormack-Lehane Grade 3 + 4 with Macintosh	+	Obesity	
Limited cervical spine mobility	+	Older age	+
Limited mandibular protrusion	+	Male sex	+
Short thyromental distance	+	Poor dentition	+
		Limited mouth opening	+
		Limited cervical spine mobility	+
		Short thyromental distance	+
		Rotation of surgical table during case	
		Applied cricoid pressure	

*Predictors of difficult ligthwand use*		*Predictors of difficult cricothyrotomy*	

Obesity (thick neck)		Neck surface pathology (e.g. scars, radiation, inflammation, hematoma)	+
Limited cervical spine mobility	+	Deviated airway (e.g. goiter, neoplasms)	
Large tongue/epiglottis	+	Obesity (thick neck)	
		Age <8–10 years	
		Female sex	
		Limited cervical spine mobility	+
